# Linea Nigra: Case Report of a Woman With a Pregnancy-Associated Linear Streak of Cutaneous Hyperpigmentation on Her Abdomen From the Umbilicus to the Pubic Symphysis

**DOI:** 10.7759/cureus.48408

**Published:** 2023-11-06

**Authors:** Philip R Cohen

**Affiliations:** 1 Dermatology, University of California Davis Medical Center, Sacramento, USA; 2 Dermatology, Touro University California College of Osteopathic Medicine, Vallejo, USA

**Keywords:** women, prostate, pregnancy, nigra, men, linea, hyperpigmentation, estrogen, cancer, alba

## Abstract

Linea nigra is a distinctive presentation of asymptomatic cutaneous hyperpigmentation on the abdomen that usually extends from the umbilicus to the pubic symphysis. It is frequently observed as a physiologic change associated with pregnancy. A primigravida 19-year-old woman began to develop skin darkening during week 24 of gestation. She delivered a healthy infant. Three months postpartum, the hyperpigmentation had not resolved. After the benign characteristics of her cutaneous hyperpigmentation were explained, the patient decided to clinically monitor the dark linear streak. Similar to this patient, clinical studies of pregnant women have observed the incidence of pregnancy-associated linea nigra to range from 32% to 92%; in contrast to this woman, partial or complete spontaneous resolution of the skin darkening commonly occurs after delivery of the newborn. During gestation, the development of linea nigra has been postulated to be caused by elevated estrogen, progesterone, and/or melanocyte-stimulating hormone levels. Linea nigra is not restricted to gestational females; it has also been noted in newborns and children. In addition, it has also been observed in men who had either benign prostate hyperplasia or prostate cancer. In summary, linea nigra is often an acquired longitudinal streak of benign cutaneous hyperpigmentation on the abdomen; when linea nigra is pregnancy-associated, the skin darkening often partially or completely resolves, spontaneously after delivery.

## Introduction

There are several physiologic changes that may occur during pregnancy. Localized or diffuse hyperpigmentation is commonly observed in approximately 90% of women. This includes not only facial melasma but also hyperpigmentation of the anus, areola, axillae, genitalia, neck, nipple, perineum, and medial thighs; in addition, scars can darken. Pregnancy-associated linear hyperpigmentation from the umbilicus to the pubic symphysis is referred to as linea nigra; in some women, it presents as darkening of a white longitudinal line (linea alba) at the site [1-4].

There are several linear lesions that can be encountered during the evaluation of dermatologic conditions. Linear hyperpigmentation is a unique anatomical configuration that can be associated with benign and malignant conditions. Linea nigra is a presentation of linear hyperpigmentation that is most frequently observed in pregnant women [3,5-7].

A 19-year-old postpartum woman presented for the evaluation of an asymptomatic dark flat linear streak of hyperpigmentation that recently developed late during the second trimester of her first pregnancy. The diagnosis of linea nigra was established based on clinical history and lesion morphology. The pigmentation persisted for several months after the delivery of a normal infant. The features of linea nigra are reviewed.

## Case presentation

A healthy 19-year-old Spanish woman presented three months following the delivery of a healthy infant for the evaluation of persistent asymptomatic hyperpigmentation that had developed during her first pregnancy. She initially noticed darkening of her skin beginning during week 24 of gestation. The skin discoloration was longitudinal; there had not been a white linear line previously at the site.

**Figure 1:**
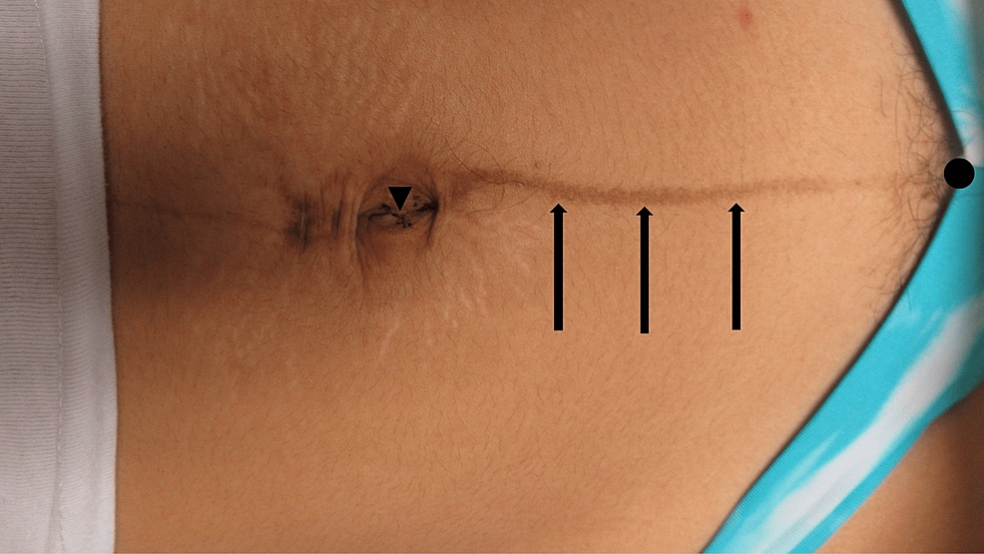
Linea nigra presenting as a linear streak of hyperpigmentation A linear streak of asymptomatic skin darkening developed on the abdomen of a 19-year-old woman beginning during week 24 of gestation. The cutaneous hyperpigmentation (black arrows) extended from her umbilicus (black triangle) to her pubic symphysis (black circle). There had been no spontaneous lightening of the hyperpigmentation by three months after the delivery of a healthy infant.

Cutaneous examination showed linear hyperpigmentation extending from her umbilicus to her pubic symphysis (Figure 1); in addition, hyperpigmentation both above and within the umbilicus was noted. Correlation of the patient's clinical history and the location of the linear streak on her abdomen established a diagnosis of linea nigra. The benign characteristics of her cutaneous hyperpigmentation were discussed. Although her newly developed skin darkening had not lightened during the first three months following delivery, the possibility of eventual spontaneous partial or complete lightening of the darkened skin was discussed. She agreed to clinically monitor the linear streak of hyperpigmentation.

## Discussion

Linea nigra is a physiologic change of pregnancy; in this setting, it has been referred to as linea gravidarum [8]. The hyperpigmentation predominantly appears as a macular longitudinal band of pigment that begins at the umbilicus and progresses to the pubic symphysis [5]. Less commonly, it has been observed to extend from the xiphoid process to the pubic symphysis [3,9].

In some of the patients, linea nigra appears de novo. The woman described in this report did not have a history of a prior linear white band at the site and noted that her skin hyperpigmentation began at the end of the second trimester of her first pregnancy. In other individuals, linea nigra develops from progressive hyperpigmentation of linea alba [3,5].

Linea alba is a linear white line that may be present extending from the xiphoid process of the sternum to the superior pubic ligament. It is formed by the fusing of the aponeuroses that are attached to the medial borders of the muscle sheaths in the midline of the body. Linea alba results from the fusing of the aponeuroses of the rectus abdominis muscles on the abdominal wall [10].

Pregnancy-associated linea nigra is rather common (Table 1) [8,11-14]. A summary of several studies shows that the incidence of linea nigra was 53%; individual studies showed the percent of pregnant women with linea nigra ranged from 32% to 92%. It has been postulated that elevated estrogen, progesterone, and melanocyte-stimulating hormone levels account for the development of linea nigra during gestation. In some of these individuals, the hyperpigmentation eventually either partially or completely fades after delivery; the young woman in this report had not experienced any lightening of the new hyperpigmentation three months after she had delivered her child.

**Table 1:** Observation of linea nigra in pregnant women Ref: references

Year	Nationality of women	Number of women with linea nigra	Total number of women	Percent of women with linea nigra	Ref
1994	French	45	60	75	[11]
1998	Pakistani	45	140	32	[12]
2005	Nigerian	46	50	92	[8]
2010	Tunisian	48	100	48	[13]
2011	Indian	1056	2000	53	[14]
Total		1240	2350	53	

The appearance of linea nigra is not restricted to pregnant women. It has been observed in the neonatal period during the evaluation of newborns during the first four weeks of extrauterine life. In a study of 1000 Indian children, 445 (44.5%) of the newborns had linea nigra [15].

Another study of 1550 Nigerians included three groups of females and males, each of 250 persons, ranging in age from 0 to 15 years, 16 to 30 years, and 31 to more years; an additional group of 50 nonpregnant women aged 27-44 years were compared to 50 of the pregnant women aged 27-44 years. Linea nigra was most common between the ages of 16 and 30 years (47.3%), followed by 0 and 15 years (31.4%) and over 31 years. For all the age groups, linea nigra was more frequently observed in women than men. Indeed, the incidence of linea nigra was 92% in pregnant women aged 27-44 years [8].

In addition, the study of 1550 Nigerians demonstrated that linea nigra was present in 80% of the men older than 50 years of age who also had either benign prostatic hyperplasia or prostate cancer [8,16]. A second study evaluating age, gynecomastia, pelvic hair distribution, and linea nigra was performed that included 50 men with benign prostate hyperplasia, 25 men with prostate cancer, and 45 men with urethral stricture and other medical problems. Linea nigra was observed in 13 (26%) of the men with benign prostate hyperplasia, 12 (48%) of the men with prostate cancer, and 8 (3.6%) of the men in the control group. After analyzing the data, using multinomial logit regression, the researchers concluded that the presence of linea nigra alone could not be used as a clinical feature, to avoid diagnostic procedures, to differentiate men with benign prostate hyperplasia from men with prostate cancer [16].

## Conclusions

Linea nigra is a unique manifestation of skin darkening on the abdomen. The distinctive cutaneous hyperpigmentation usually presents as a longitudinal streak from the umbilicus to the pubic symphysis. Pregnancy-associated linea nigra that developed during the latter part of the second trimester of a 19-year-old woman's first pregnancy is described in this report. The hyperpigmentation of her skin persisted for three months after she delivered a healthy infant. Linea nigra is commonly observed as a physiologic change associated with pregnancy. Often, after delivery of the newborn, partial or complete spontaneous resolution of the skin darkening is observed. In addition to pregnant women, linea nigra has not only been noted in newborns and children. It has also been observed in men who have either benign prostate hyperplasia or prostate cancer; however, the presence of this clinical feature alone cannot be used as a screening evaluation to differentiate men with benign prostate hyperplasia from men with prostate cancer. In conclusion, linea nigra is an asymptomatic, benign linear streak of skin darkening on the abdomen; when associated with pregnancy, partial lightening or complete disappearance of the cutaneous hyperpigmentation often occurs, without treatment, during the postpartum period.
